# K-Nearest Neighbors for Anomaly Detection and Predictive Maintenance in Water Pumping Systems

**DOI:** 10.3390/s25113532

**Published:** 2025-06-04

**Authors:** João Pablo Santos da Silva, André Laurindo Maitelli

**Affiliations:** Computer Engineering and Automation Department, Federal University of Rio Grande do Norte, 3000 Senador Salgado Filho Avenue, Natal 59078-970, RN, Brazil

**Keywords:** hydraulic anomalies, water pumping systems, machine learning

## Abstract

The importance of maintenance activities for improving the quality of water sources and guaranteeing a steady supply of water has increased significantly because of current social concerns. Water supply pipe corrosion is an issue that can cause leaks and lower water quality. The identification of hydraulic anomalies in water pumping systems is the subject of this project. A database was created of data acquired from a water supply network with pipes of various lengths and sizes. In hydraulic systems, sensor meters are mounted at various sites with distinct physical features, pipe sizes, and vital supply points. The input parameters used for a model are the sensor parameters, and the model analyzes the correlation between the input parameters (sensors) and determines which parameters are the most important, deciding on the output of the model, and thereby building the simplest model, which requires the least input parameters and gives the most accurate prediction results. In this project, using on the input signal from the sensors, the k-nearest neighbors machine learning algorithm was used to correlate/predict whether the pump was shut down (broken) for a certain period of time.

## 1. Introduction

Pump systems are integral to the operation of power plants, serving as the primary means of electricity generation. Notable sources of power include thermal, hydroelectric, and nuclear energy. It is essential to ensure that these systems are maintained in an optimal condition, in order to guarantee a continuous power supply. In the event of a pump failure, there is a potential reduction in power generation, which may ultimately result in a complete shutdown. Such an occurrence could be averted if the failure could be predicted in advance, allowing for the implementation of measures to prevent significant economic losses, as proposed. The challenging process of manually identifying and assessing issues can lead to inaccuracies when personnel are required to traverse extensive lengths of pipelines for inspection purposes. The deployment of sensors can facilitate the acquisition of data pertaining to flow density, volume, load capacity, pressure, vibration, temperature, and other relevant parameters. In such scenarios, the application of machine learning (ML) methodologies can prove advantageous, as they can replace time-consuming and ineffective manual detection techniques. This approach can consequently facilitate a more expedient and effective hydraulic resource crisis response, as well as the identification of pump failure.

An urban water supply network system can be conceptualized as a mobile data carrier, transmitting information regarding pipe flow rate, nodal pressure, and water quality. This is performed in a manner that ensures satisfaction of energy balance, pressure balance, and water quality balance conditions [1]. Automatic detection of such faults using ML can make the process much more convenient and produce more accurate results [2]. Various studies were classified by [3] as mechanisms for estimating water losses, which is generally carried out using the water balance, and the identification and location of water leaks in distribution networks. Infrared photographs [4], acoustic devices [5], induced pressure waves [6], and robots in pipelines [7] have recently been tested for identifying leaks in previously defined areas of a distribution network.

Computer-based models can be designed with two fundamental objectives: to identify the occurrence of a leak in a system, and to record the approximate location of the leak. The process of leak identification entails the analysis of physical measurements, including the level of water reservoirs, the pressure and flow of pipe sections, and water demands, in order to ascertain the time interval in which the leak occurred.

Identification or localization methods are employed in conjunction with modeling hydraulics, which is achieved through optimization techniques, including genetic algorithms and search space reduction [8,9], or with algorithms based on pressure data with high temporal resolution, such as the K-nearest neighbors (KNN) algorithm [10], convolutions using neural networks [11,12,13], long short-term Memory (LSTM) neural networks [14], and Bayesian classifiers [15].

Leak detection techniques can be classified into two main categories: internal and external. External techniques include radar detection, hydrophones, and acoustic logging, which are time-consuming and have limited applicability due to their localized nature. Internal techniques, on the other hand, can be grouped into four principal categories: transient-based methods, calibration-based approaches, data-driven methods, and model-based techniques [2]. The fundamental premise of data-driven methodologies is the extraction of reliable information from a substantial corpus of data samples, with the objective of constructing data-driven models [16]. A machine learning technique based on statistical learning theory, using a support vector machine (SVM) model, was developed to address the issue of limited data samples [17,18,19]. A model-driven decision support system was developed, based on the integration of a calibrated hydraulic model of a water network with a model-based approach to leak location, which made use of online telemetry data [20].

The detection of hydraulic anomalies, with particular emphasis on the identification of leaks in pipes, can be advanced through two distinct approaches: one based on the use of specialized hardware, and the other based on the implementation of sophisticated software algorithms [21]. The absence of formalized documentation and the non-linearity of the differential equations that govern the majority of pressure and flow behaviors in pumping and water transport systems present significant challenges in identifying and quantifying anomalies. This, in turn, contributes to a lack of comprehensive knowledge regarding hydraulic systems. The position of leaks and the estimation of pressures and flows generated by water loss mean that there is no simple deterministic method. Therefore, the established polynomial problem presents a significant computational burden, making it challenging to develop a computational routine for real-time operation [22].

Accordingly, comparative estimates consider flow equations, mass conservation equations, and energy conservation equations. Subsequently, volumetric knowledge of the mass and location of a leak is directly dependent on the behavior of the hydraulic gradient within the hydraulic system. When a leak occurs, there is a change in velocity within the system. Therefore, if multiple leaks are present in a hydraulic system formed of different meshes, a highly complex hydraulic problem is encountered, due to the sensitivity of the input variables.

The objective of this proposal was to utilize machine learning (ML) algorithms to identify patterns and establish predictive maintenance strategies based on an in-depth examination of the sensor data provided. The time-series analysis approach provided a general framework for understanding the underlying processes. The primary objectives were to reduce the frequency of false alarms and instances of missed detections for leakage events and to examine the potential advantages of data mining based on time series data for leak detection. The K-nearest neighbor (KNN) algorithm is relatively straightforward to comprehend and implement. The algorithm is capable of classifying non-linear datasets effectively, as it does not rely on any assumptions about the shape of the data. Furthermore, the KNN algorithm has a minimal number of configuration settings, which contributes to its ease of use. This work employed two unsupervised learning techniques, namely principal component analysis (PCA) for feature extraction and dimensionality reduction, and K-means clusters. Following the assessment of the initial architecture’s performance using imbalanced data and the testing of several network hyperparameters, an overview and summary will be provided.

## 2. Materials and Methods

Water pump systems facilitate the efficient transportation of water from its source to residential, commercial, industrial, and agricultural areas, thereby performing a crucial role in the provision of a consistent and reliable water supply. These systems have a pivotal role in ensuring the provision of essential services and the promotion of economic growth by supporting communities, agriculture, and industry. Disruption of the water supply is a significant concern when water pump systems malfunction, as this can lead to shortages, consumer inconvenience, and adverse effects on other industries that rely on a consistent water supply. The maintenance of water pump systems necessitates regular assessments, repairs, and modifications, to ensure optimal performance, prevent malfunctions, and enhance equipment durability.

In the contemporary competitive and ever-changing global environment, the optimization of maintenance strategies through the utilization of data is of paramount importance. In the contemporary operating environment, which is characterized by dynamism, it is imperative to pursue cost reduction, risk mitigation, and performance enhancement, in order to maintain a competitive advantage. The capacity to discern irregularities can facilitate enhanced maintenance strategy formulation, which represents the objective of this study. The dataset in question pertains to a water pump situated in a municipality. Over the preceding year, seven system failures were documented. These errors resulted in a significant predicament. The rationale for not disclosing the specifications of the sensors and pumps comes from non-disclosure agreements (NDAs), which are in place to safeguard the business.

The pump-related sensor data were obtained from Amazon Web Services. The dataset comprises a timestamp, data from 52 sensors, and the status of the machine in question. The sensor data were recorded at a frequency of one minute, and the corresponding machine status is also provided. The data frame indicates that there are 55 columns and 220,320 recordings. Furthermore, the measurements are presented in different scales. The data are divided into three categories: a timestamp, data from 52 sensors, and one machine status (target/results) column. The pump system provides water for a small area situated remotely from a larger town. Over the course of a year, there were seven documented instances of system failure. These failures resulted in significant challenges for numerous individuals and also precipitated serious living difficulties for some families.

Extraction–transformation–loading (ETL) tools are pieces of software that are responsible for the extraction of data from various sources, as well as their cleaning, customization, reformatting, integration, and insertion into a data warehouse. The construction of the ETL process is potentially one of the most significant tasks associated with the development of a data warehouse. It is a complex, time-consuming process that consumes the majority of data warehouse project resources, including costs and implementation efforts [23]. Exploratory data analysis (EDA) is a method used by researchers to gain insight into the characteristics of primary datasets with respect to various statistical measures. An examination of the distribution, outliers, and anomalies within the data is essential for a more comprehensive understanding of the data and for identifying areas within datasets that require further investigation [24].

EDA [25] represents a fundamental procedure that employs statistical techniques and graphical representations, with the objective of deriving insights from data [26]. EDA is a valuable tool for identifying hidden patterns and correlations among attributes in data, as well as for formulating and validating hypotheses derived from that data [27]. Moreover, the deployment of data for the purpose of training predictive models [28] for the formulation of strategic business decisions necessitates the availability of data exploration tools that can facilitate the accurate analysis of complex multivariate relationships [29] in datasets, despite the constraints on available analytical expertise.

**Figure 1:**
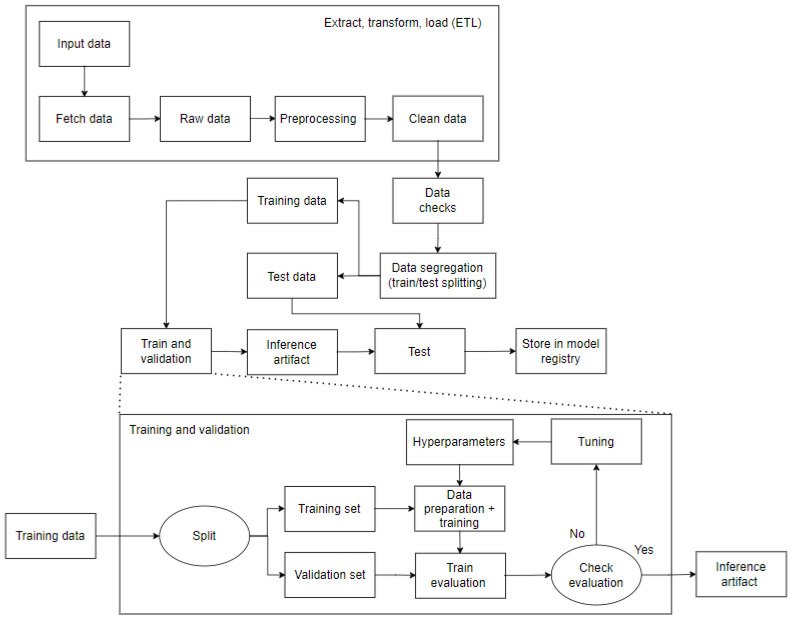
Pipeline used in implementation.

In the context of high-dimensional feature spaces, certain qualities may be superfluous and therefore unnecessary. It is of paramount importance to eliminate these superfluous or insignificant characteristics in order to achieve efficiency. The objective of feature selection is to identify a small subset of pertinent qualities from the original features, thereby enhancing the effectiveness of classification algorithms. Improved learning outcomes, such as increased learning accuracy and reduced computing expense, can result from feature selection. The project was carried out in accordance with the procedures outlined in the workflow produced and made accessible in Figure 1. The pipeline steps are depicted in the figure below.

**Figure 2:**
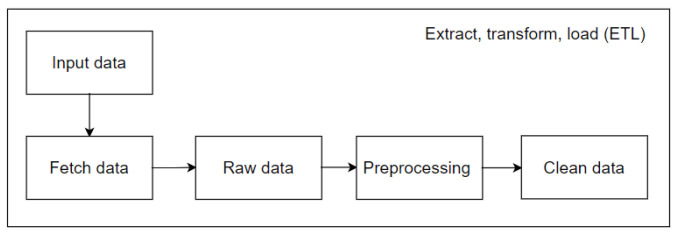
ETL methodology applied to the project.

The initial step, and arguably the most crucial, is to generate data that are suitable for model training. As illustrated in Figure 2, this stage of the process entails a number of activities, including an exploration of the database to gain insight into its structure and content, the replacement or removal of corrupted data or data that are unsuitable for the model, an analysis of the database’s dimensionality, and an examination of the relationships between features.

The subsequent stage of data processing represents a further advancement. An analysis demonstrated that the selected dataset did not contain any duplicate lines and that no additional issues required correction. Consequently, the project proceeded to the subsequent phase. It was essential to verify the accuracy of the dataset prior to training the model. This was followed by a phase of data verification. The dataset comprised three types of data, which were confirmed during the exploratory data analysis process: integer, binary, and category. Consequently, categorical data had to be converted into numeric variables prior to training. The one-hot encoding method was applied to achieve this. As a result, duplicate features were eliminated and new ones were added to the training and testing datasets.

**Figure 3:**
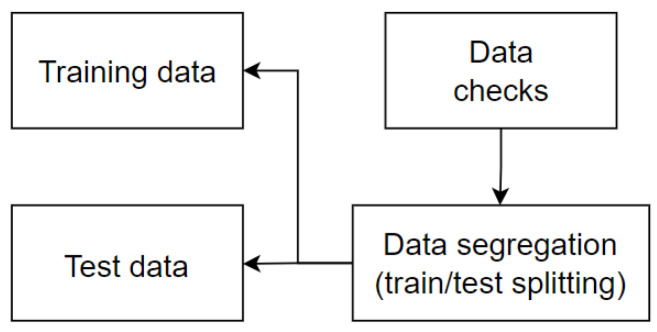
Data separation methodology.

Subsequently, it was necessary to transform all of the knowledge gained from the data during the EDA process, including the earlier processing involved in completing any missing values that the extract, transform, and load process had missed. The dataset was divided into multiple sections during the data segregation phase. Here, a 80/20 ratio was applied. Specifically, 80% of the dataset was designated for the model training and validation, which was saved as a train.CSV artifact. The remaining 20% was set aside for the test, as a model testing.CSV artifact. Notably, the training dataset was split into training and validation sets while it was still in the segregation stage, according to Figure 3.

## 3. Results

One of the most challenging aspects of utilizing machine learning is the effective deployment of the comprehensive range of models developed in training and scenario simulation environments in actual operational contexts. Despite a model’s impressive performance, its true efficacy can only be fully realized when deployed in the real world or in production environments. This necessitates mastery of the requisite technology.

Exploratory data analysis is a crucial step in working with sensor data. In this study, the data were examined in their entirety prior to the division into training and test sets. The EDA section employed visualizations and initiates preliminary data preprocessing steps, as evidenced by the extensive dataset and the removal of empty columns, as illustrated in Table 1. The data were recorded in one-minute intervals, as indicated by the timestamp. The sensor data were recorded in float32 format with varying amplitudes and presented in the yyyy-MM-dd HH:mm:ss format.

In EDA, it is crucial to gain a comprehensive understanding of the data to be analyzed, in order to develop an effective solution strategy. Given that the data were time-series data and based on clustering models, it was decided to split the data after a preliminary examination. Additionally, it was observed that the data had three distinct labels, necessitating the representation of all labels in the testing set. Consequently, the decision was made to split the data based on these observations.

A cursory examination of the data was necessary to ascertain the results, which were then utilized to establish prediction targets. The target data were continuous and presented in a readily comprehensible format. A comprehensive overview of the sensor data and the associated labels is provided. The raw data statistics are displayed as follows: normal, broken, and recovering. Moreover, a complete set of target information is displayed for each sensor every minute. After enumerating the values within each class, it was determined that the majority class was “Normal”, as the machine was operating within normal parameters, while the classes “Recovering” and “Broken” constituted a minority of the dataset. The “Normal” class represented 93.4% of the dataset, comprising 205,836 records. The “Recovering” class accounted for 6.57% of the dataset, comprising 14,477 records. The “Broken” class comprised only seven records. At the following timestamps, reports of distribution network disruptions were documented: 17,155, 24,510, 69,318, 77,790, 128,040, 141,131, and 166,440. The failure events occurred in April (twice), May (twice), June (once), and July (twice) in 2018. Notably, the aforementioned incidents occurred on the following dates and times: 12 April 2018, at 21:55:00, Thursday (timestamp 17,155); 18 April 2018, at 00:30:00, Wednesday (timestamp 24,510); and 19 May 2018. Additionally, the following instances of failure were recorded: 12 April 2018, 21:55:00, Thursday (timestamp 17,155); 18 April 2018, 00:30:00, Wednesday (timestamp 24,510); 19 May 2018, 03:18:00, Saturday (timestamp 69,318); 25 May 2018, 00:30:00, Friday (timestamp 77,790); 28 June 2018, 22:00:00, Thursday (timestamp 128,040). The remaining instances occurred at the following timestamps: 8 July 2018, 00:11:00, Sunday (timestamp 141,131); and 25 July 2018, 14:00:00, Wednesday (timestamp 166,440).

This is one method for obtaining an understanding of the distribution of data and for identifying instances of missing values. Upon analysis of the target, it became evident that faulty sections were not grouped together but rather dispersed throughout the entirety of the dataset. This observation is particularly noteworthy in the context of data segmentation, specifically with regard to the delineation of training, test, and validation sets. Furthermore, the recovering class was observed to follow the broken class, which was a promising indication that the reduced number of entries for the broken class did not pose a significant challenge. This was because the recovering class could be utilized to predict the broken class, effectively addressing the issue of limited data points. Additionally, each row in the dataset contains a single entry, which leads to a single machine process with numerous sensors and supervised learning capabilities. This is an important aspect to consider when developing a data analysis strategy.

**Figure 4:**
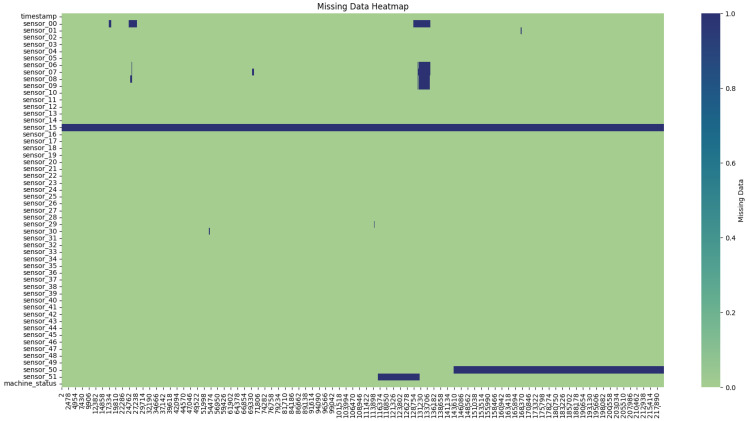
Missing data heat map.

The subsequent phase of the process entailed an examination of the quality of the sensor data, with the objective of repairing the data in a manner that rendered them fit for utilization in training. The initial step was to identify the English acronym for “Not a Number” (NaN) values. This is typically indicative of a sensor that did not transmit any data for a number of reasons, including inadequate maintenance or an issue with the signal itself. It is crucial to distinguish between NaN values and zero values. While zero values may signify a lack of data, the presence of numerous zeros in a dataset indicates a sparse dataset. Upon examination of the data, it became evident that only a few sensors exhibited NaNs, with some sensors displaying a greater prevalence of empty values than others, as illustrated in Figure 4. Furthermore, the problematic data were concentrated in specific regions, allowing for the meticulous filling of NaNs.

From this information, it can be seen that there were a number of instances where data were absent. For example, sensor 15 had no values, sensor 50 had 34.95% of its data missing, and sensors 00 and 51, which were both missing close to 6–7% of their data, also exhibited this pattern. After removing the prior columns, a missing data heat map was generated to assess the remaining missing values.

It was observed that a significant portion of the dataset exhibited a pattern of simultaneous sensor failure (Figure 4). This could have been caused by a number of factors, including weather, electrical or mechanical malfunctions, maintenance, or other potential reasons. As this could be indicative of the system being offline, the proposed model retained these instances and the data were subsequently omitted. There were two potential courses of action: either the remaining rows with missing values could be deleted, or all NaNs could be removed from the data. However, the latter approach would have resulted in the loss of approximately 77,000 timesteps, representing a reduction of about 35%. Consequently, it was advisable to attempt the removal of as many NaNs as possible, sensor by sensor.

The sensors with the highest concentration of NaNs were sensors 50 and 51. Firstly, sensor 50 malfunctioned at a specific point in time. Two potential courses of action emerged: the complete removal of sensor 50, or the removal of all data from all sensors from time step 140,000 onward. Secondly, both sensors exhibited a similar amplitude and range of values. Moreover, the yellow-marked sections were highly analogous. In other sensors (see Figure 4), the decline at mark 140,000 was evident in the majority of sensors. Sensor 51 also appeared to demonstrate this decline immediately following its data gap. Consequently, the decision was taken to utilize sensor 50 to repair sensor 51.

**Figure 5:**
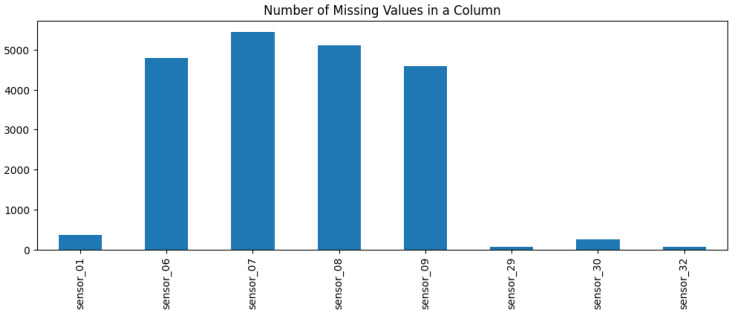
Number of missing values in a column.

**Figure 6:**
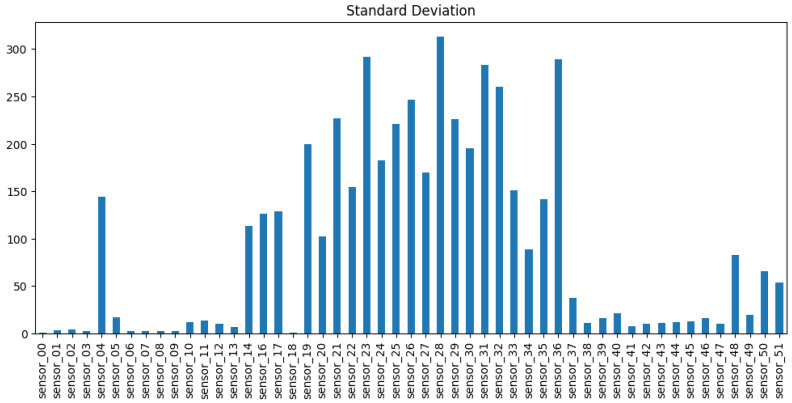
Standard deviation for sensors.

Following the repair, it was evident that sensors 01, 29, 30, 32, and the sensors situated between 06 and 09 exhibited the highest incidence of NaNs, as illustrated in Figure 5. Accordingly, the subsequent step was to examine the standard deviation and variance, which illustrate the extent to which a signal deviates from the mean. This indicates whether a signal is undergoing any kind of movement, and it is therefore essential to identify any trends or changes in the classification. In the highlighted section of Figure 6, the sensors 01, 06–09 do not exhibit a high standard deviation. Consequently, they could be filled with a limit set to 30, representing a maximum of 30 consecutive NaN values.

Each column was missing approximately 2% or less of its data, which could be filled in. Prior to any data manipulation, the data were split into training and testing data using an 80/20 split. As they are time series data and the model is designed to predict new data, the last 20% of the data were used for testing. However, it is of great importance to note that the dataset contained seven instances of failure, which were broken instances.

As illustrated in Figures below, the sensors in question typically exhibited a normal recording. The subsequent group represents the recovery phase, which is a logical consequence of the machine suffering an interruption and subsequently recovering. It is notable that only 0.004% of the data had a broken status, which was an acceptable percentage, given that a machine is expected to remain operational for a long period of time. It appears that some sensors exhibited similar behavior despite different scales, and thus it may be beneficial to group sensors based on observed behavior.

**Figure 7:**
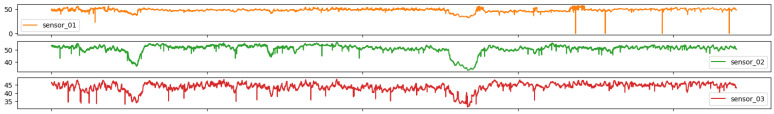
Time series sensors—Group 01.

Figure 7 illustrates the impact of hydraulic variations in the distribution network on the sensor signal, particularly the influence of medium-frequency components.

**Figure 8:**
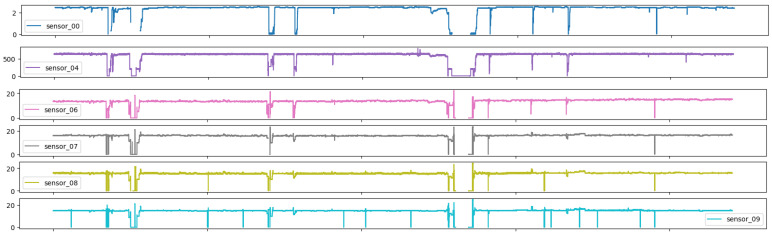
Time series sensors—Group 02.

Figure 8 illustrates the loss of signal observed shortly after the central measurement period in the six highlighted sensors. It is notable that these sensors monitored the same variable and exhibited a similar measured behavior within close ranges, with the exception of sensor 04, which had a 25× larger scale. This suggests that sensor 04 may represent the output flow of the entire system.

**Figure 9:**
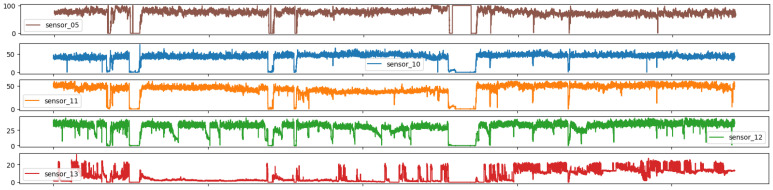
Time series sensors—Group 03.

Figure 9 illustrates the presence of pronounced high-frequency components in the signals, which suggests that the variable in question was directly linked to the water distribution pipes and was susceptible to hydraulic variations within the system, particularly with regard to the load inputs and outputs within the distribution network.

**Figure 10:**
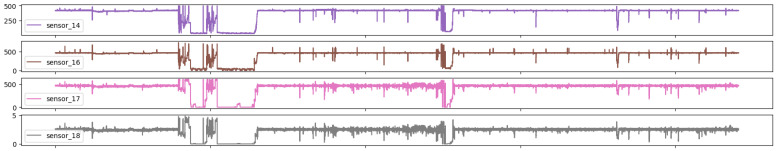
Time series sensors—Group 04.

As illustrated in Figure 10, the sensor behavior exhibited a hydraulic anomaly that manifested subsequent to the system’s operational commencement. This anomaly demonstrated the potential to precipitate abrupt fluctuations proximate to the system’s maximum and minimum variable scales.

**Figure 11:**
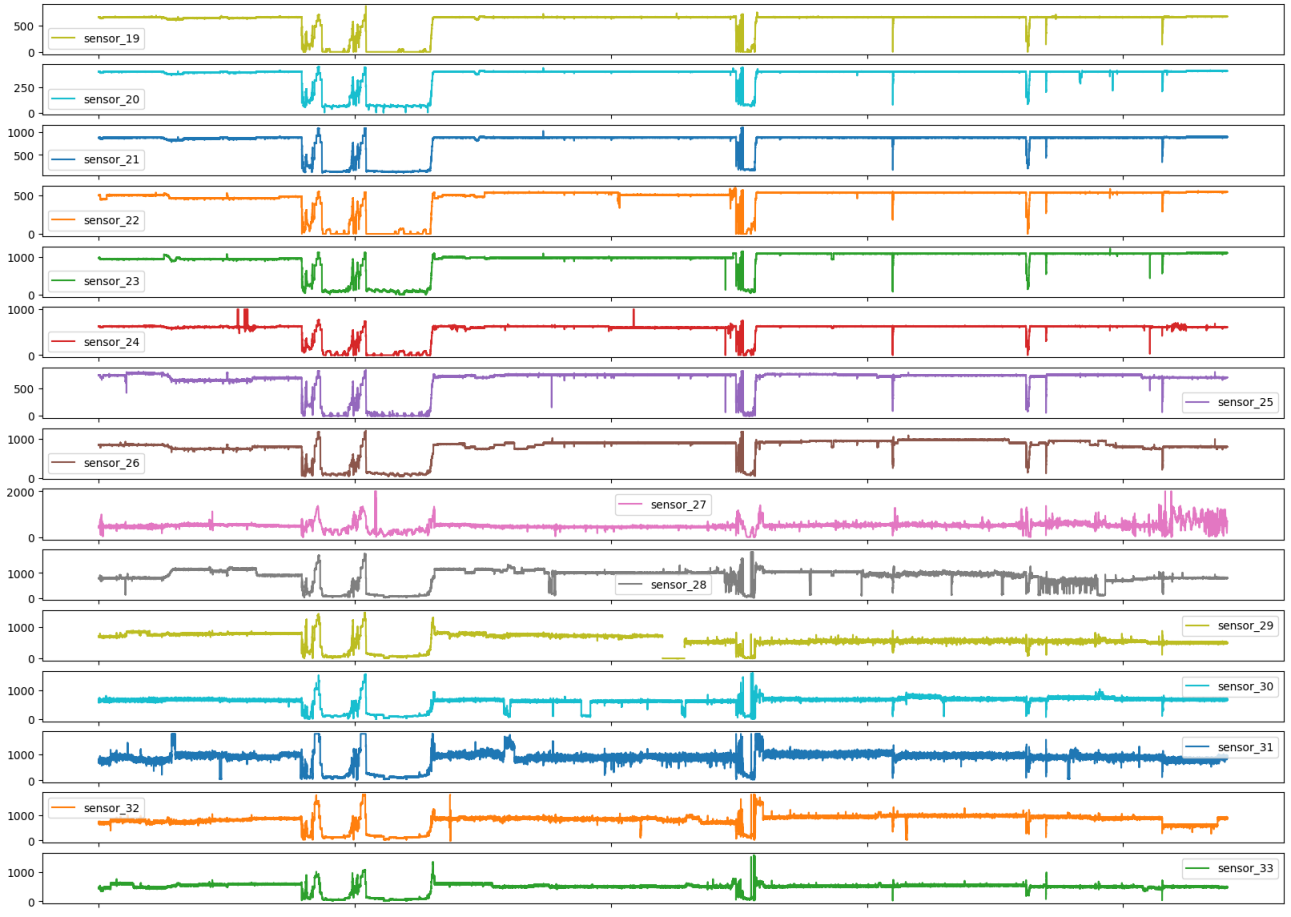
Time series sensors—Group 05.

As can be observed in Figure 11, the sensors demonstrated a smooth hydraulic behavior, exhibiting a gradual response to anomalies in the distribution network.

**Figure 12:**
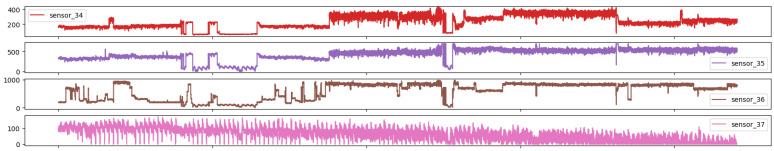
Time series sensors—Group 06.

Figure 12 illustrates the grouping of sensors with more distinct behaviors when analyzed temporally, with a notable emphasis on sensor 37, which exhibited pronounced oscillatory characteristics and high frequency bands.

**Figure 13:**
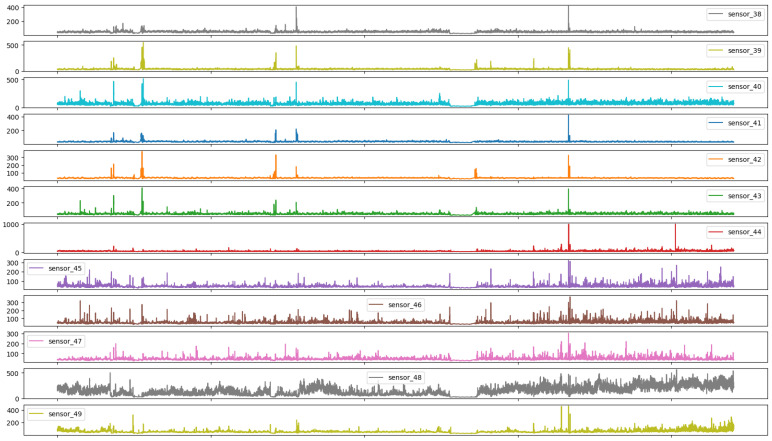
Time series sensors—Group 07.

Group 07 sensors, as presented in Figure 13, showed a behavior analogous to that of impulse responses, thereby underscoring the responsiveness of the sensors employed, which even at disparate amplitudes manifested analogous temporal responses.

**Figure 14:**
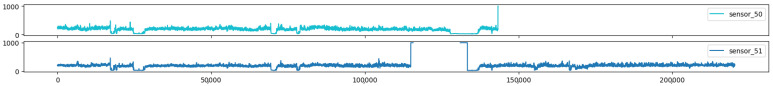
Time series sensors—Group 08.

Group 08, as represented in Figure 14, had two sensors that exhibited a considerable reduction in measurement range. Consequently, they were subjected to a comprehensive examination to ascertain potential solutions to this issue.

**Figure 15:**
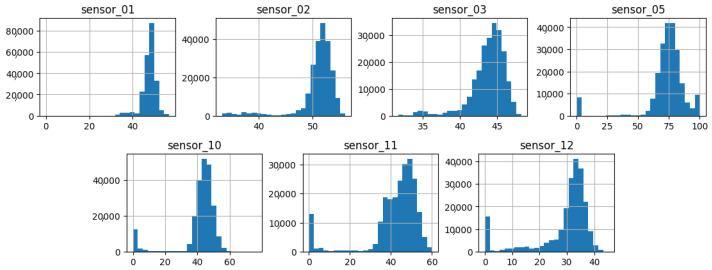
Sensor histograms—Group 01.

As illustrated in Figure 7, Figure 8, Figure 9, Figure 10, Figure 11, Figure 12, Figure 13 and Figure 14, it is evident that a considerable number of the plotted data points exhibit pronounced fluctuations around the x-marker, with frequencies reaching 40,000–65,000 and 130,000 instances, respectively. As illustrated in Figure 15, the sensor histograms highlight instances where certain sensors exhibited a notable decline in value, while others demonstrated an increase. Additionally, there were instances where sensors that typically maintain a consistent value exhibited a significant variation. These observations suggest that the machine may have encountered a failure and was undergoing a recovery process. This could serve as an indicator for predicting potential failures. This brings us to the next task: addressing the issue of missing data.

**Figure 16:**
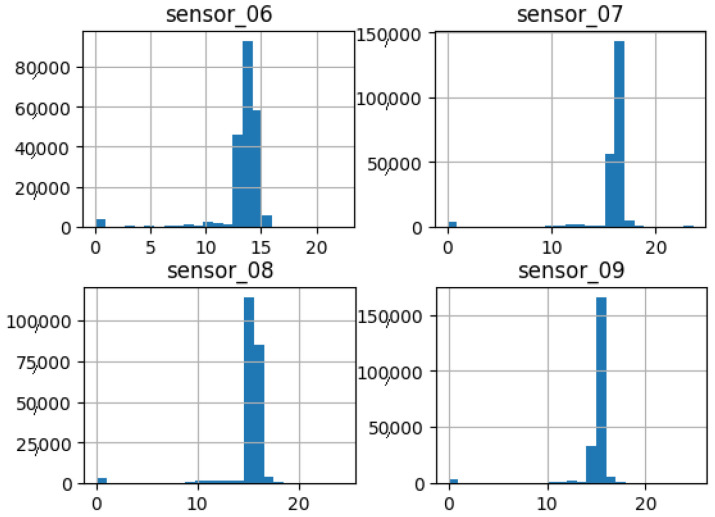
Sensor histograms—Group 02.

**Figure 17:**
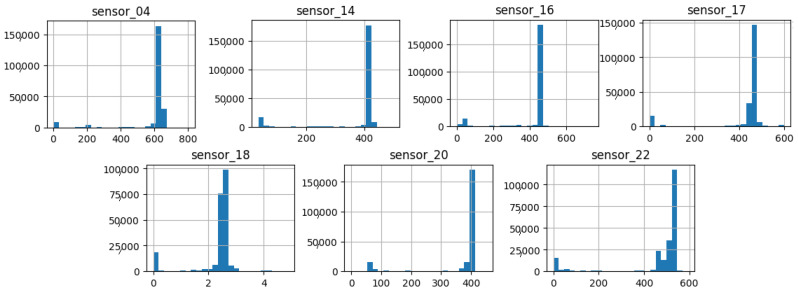
Sensor histograms—Group 03.

**Figure 18:**
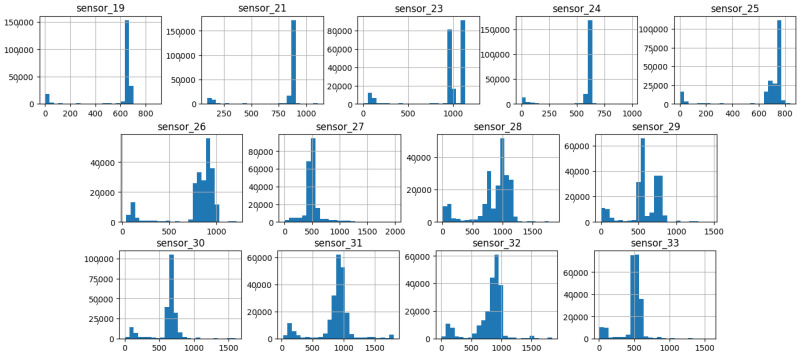
Sensor histograms—Group 04.

**Figure 19:**
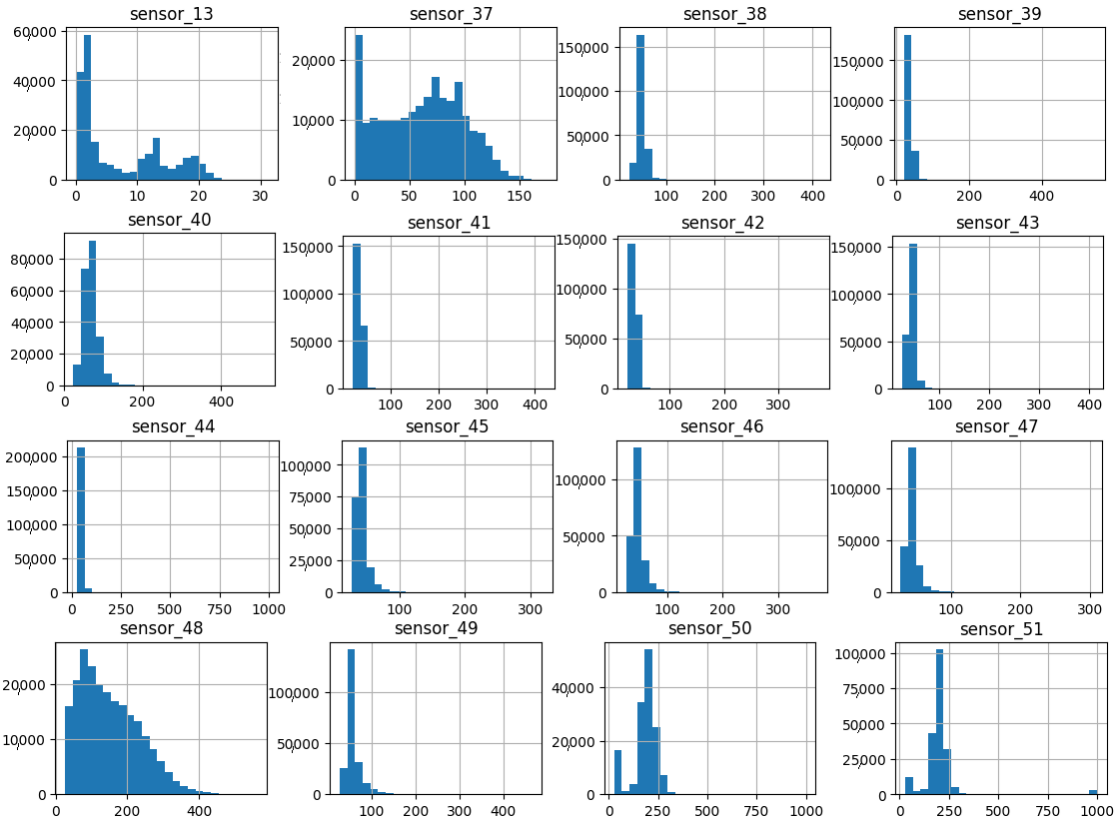
Sensor histograms—Group 05.

**Figure 20:**
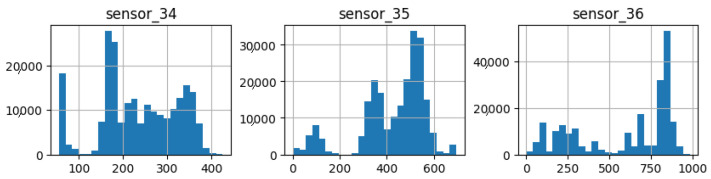
Sensor histograms—Group 06.

As shown in Figure 15, Figure 16, Figure 17, Figure 18, Figure 19 and Figure 20, the presented histograms demonstrate the shape of the sensor data. The values of the sensor data are displayed on the horizontal axis, with the range of values represented by each bar. The number of data points falling within the specified range is displayed on the vertical axis. The bars illustrate the number of values for each sensor range, demonstrating that the center and the spread are distinct for each sensor. Furthermore, the data exhibit a mound-shaped distribution, which is indicative of a normal distribution. These graphs emphasize a pivotal point: whether to organize data into categories. As groupings are possible, it is recommended to create separate histograms for each group, to facilitate a more comprehensive understanding of the data.

Group 01 of sensors, as displayed in Figure 15, exhibits a normal distribution with a mean value situated between 40 and 75, contingent upon the specific sensor scale. Moreover, a standard deviation with analogous ranges and an initial peak proximate to the value of zero can be observed in sensors 5, 10, 11, and 12.

Group 02 of sensors (Figure 16) also demonstrates a normal distribution with an average value approaching 15, indicating a reduction in sensor amplitude. Additionally, a low standard deviation is observed. This suggests that the sensors exhibited high repeatability, with a diminished initial peak approaching the 0 value.

Group 03 of sensors (Figure 17) exhibits a notable elevation in the amplitude of measured values, with averages within the range of 400 to 650, with the exception of sensor 18, which displays a value approaching 3. Additionally, this group demonstrates a narrow standard deviation, indicating minimal variability in the measured variable.

Group 04 of sensors (Figure 18) exhibits elevated mean amplitudes from 500 onward, accompanied by a pronounced standard deviation and a prolonged tail extending to the initial values. This group demonstrates a variable with high sensitivity to the response of the hydraulic system.

The set of sensors in group 05 (Figure 19) exhibits an average value between 2 and 250, with an average standard deviation. Of particular interest is the long right tail that shows high values, even with low comparative averages. It is also noteworthy that despite the long group, the quantity is relatively low compared to the main range.

The three sensors in the sixth group (Figure 20) exhibited bimodal behavior and a high standard deviation, which is noteworthy. They demonstrated high repeatability across the majority of the measurement range, indicating that the measured variable was of significant importance throughout the entire range of the sensor.

**Figure 21:**
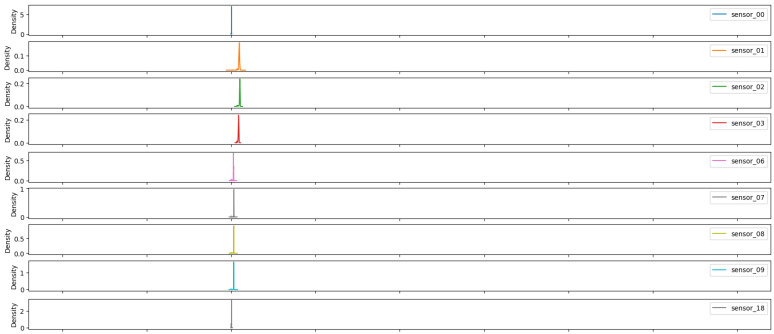
Kernel density estimation for sensors values—Group 01.

**Figure 22:**
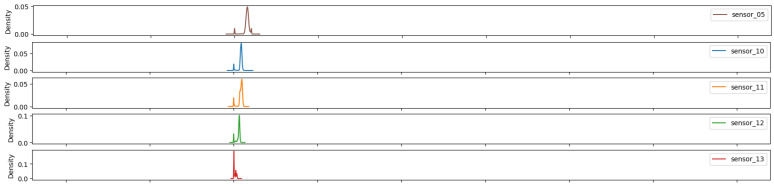
Kernel density estimation for sensors values—Group 02.

**Figure 23:**
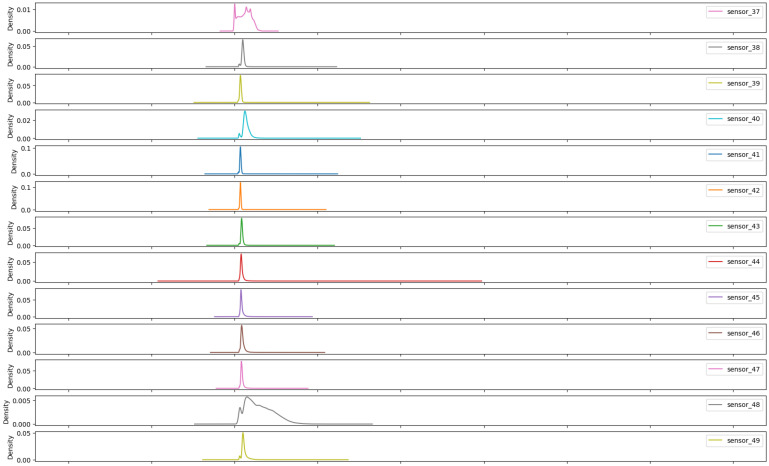
Kernel density estimation for sensors values—Group 03.

**Figure 24:**
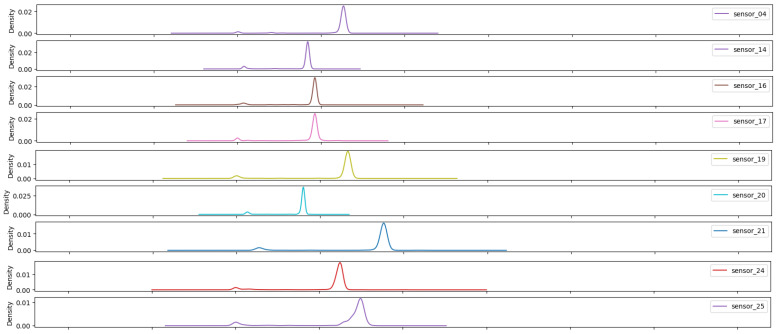
Kernel density estimation for sensor values—Group 04.

**Figure 25:**
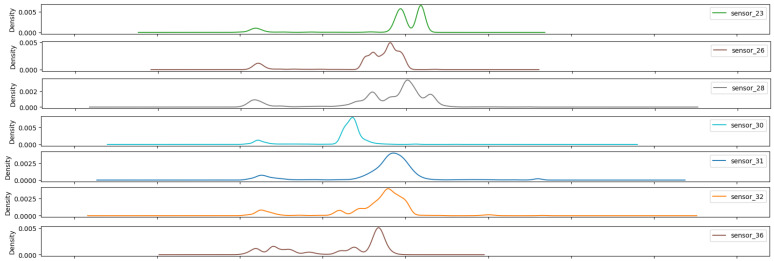
Kernel density estimation for sensor values—Group 05.

**Figure 26:**
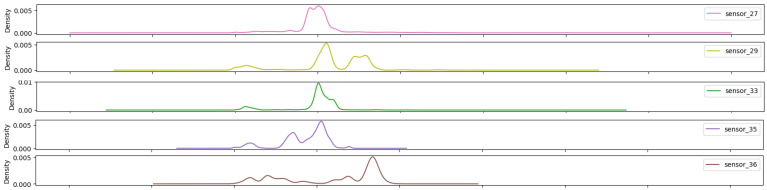
Kernel density estimation for sensors values—Group 06.

**Figure 27:**
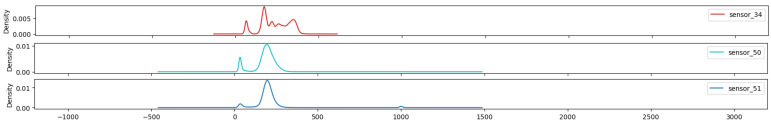
Kernel density estimation for sensors values—Group 07.

In statistics, the non-parametric method for estimating a random variable’s probability density function (PDF) is referred to as kernel density estimation (KDE). This function features automatic bandwidth determination and makes use of Gaussian kernels, which invariably reduce the amount of error in arriving at the true density. The objective of this work was to achieve adaptive multivariate kernel density estimation [30]. As previously mentioned, some sensor sets showed similar shapes (see Figure 21, Figure 22, Figure 23, Figure 24, Figure 25, Figure 26 and Figure 27).

The kernel width was a determining factor in the level of smoothing applied to the estimate and in the exclusion of outliers that deviated from the main distribution. In this group (Figure 21), a peak is observed that is both sharp and close to the value of zero, as well as narrow.

Group 02 presents a bimodal aspect (Figure 22), indicating a difference that is close to zero. However, it is notable that this group exhibits a higher peak in the range further from zero, similarly to the previous group.

The third group (Figure 23) demonstrates a statistical behavior analogous to the first, with a notable distinction being the widening of the band, which signifies a heightened variability within the measurement range.

The sensors in group 04 (Figure 24) demonstrate comparable behavior with regard to bandwidth, albeit with disparate mean values contingent on their respective measurement ranges. Moreover, they exhibit a considerable and consistent bandwidth, suggesting the reproducibility of the measured values within a narrow range.

The seven sensors that have been highlighted (Figure 25) demonstrate a wide operating band, with some of them even exhibiting bimodal behavior. These facts reveal that the sensors in group 05 are capable of measuring values across the entire measurement range.

The sensors presented (Figure 26) exhibit the highest bandwidth of the groups and multimodal characteristics, thereby demonstrating optimal sensitivity across the entire range.

The final group (Figure 27) exhibits an average bandwidth, with values that are nearly identical to the overall mean, indicating that the operational range is diminished and that the system operates within extremely narrow margins.

### 3.1. Preprocessing

In the preprocessing phase, it was advisable to extract the most pertinent data elements for input into the machine learning model. Initially, any missing data were addressed through forward propagation. Subsequently, a prevalent unsupervised learning technique, namely principal component analysis (PCA), was employed to curtail the number of sensors utilized in the subsequent stages [31]. PCA is an effective tool for feature reduction, as detailed in tabular form in Table 2.

The model initially performed preprocessing, which entailed the deletion of superfluous columns. Subsequently, it addressed the issue of missing data, which can be approached in a number of ways. In this particular case, it appeared that the missing values were associated with instances when the machine was inoperable. This is a logical assumption, given that if the machine is inaccessible, the sensor may also be inoperable, given their interconnectivity. Consequently, the model could utilize either forward or backward propagation.

Subsequently, we investigated feature extraction and dimensionality reduction through the utilization of principal component analysis (PCA), an unsupervised learning model technique. The preprocessing of the dataset entailed the following steps: the handling of missing data through forward filling, the normalization of data, and the reduction of dimensionality through PCA.

The curse of dimensionality represents a significant challenge in the field of machine learning. To address this challenge, an alternative approach was taken: the code was expanded, but the dimensionality of the data was reduced. In addition to facilitating memory management, principal component analysis can be employed to identify the most salient features for incorporation into a model, thereby enhancing its overall performance. PCA can also be utilized to determine the optimal number of components, such that the proportion of variance to be explained exceeds a specified threshold. In this example, the explained variance was set to 99%, resulting in a reduction in the number of components to 15.

**Figure 28:**
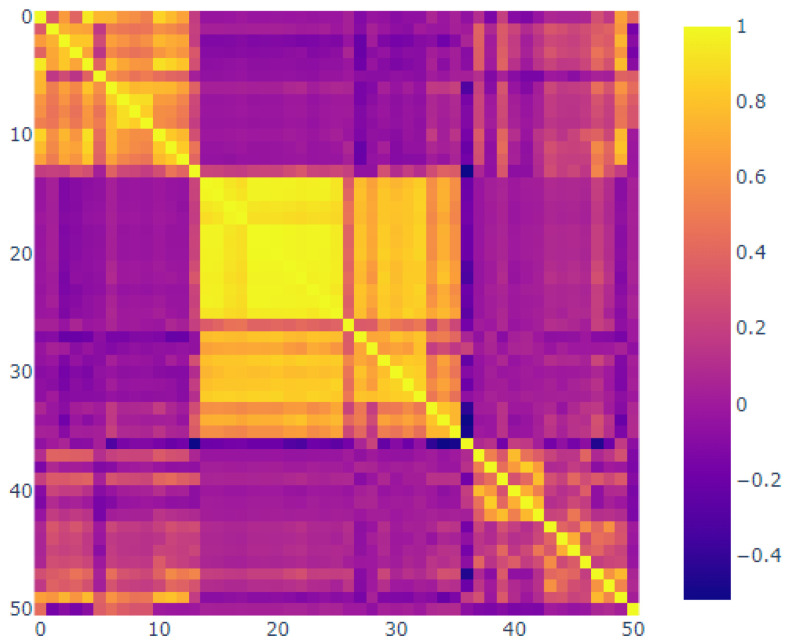
Correlation plot.

The correlation map below provides evidence for the substantial links between several of the sensors (Figure 28). It appears that at least three sets of sensors exhibited consistent behavior. The use of correlation analysis allowed for the examination of the sensors and the observation sensors, which demonstrated a strong association between sensors 14 and 26.

**Figure 29:**
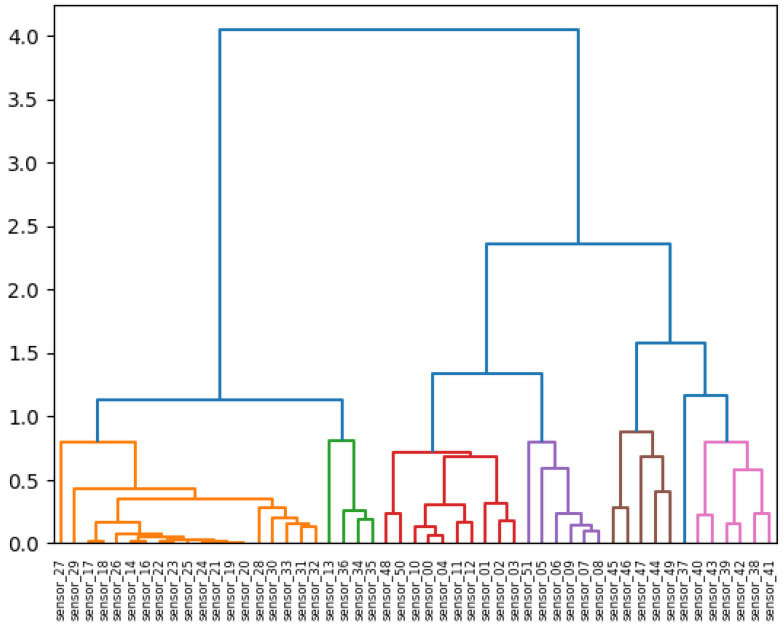
Hierarchical clustering.

In contrast, sensors 01 through 13 and 37 through 49 demonstrated minimal correlation. Additionally, sensor 37 was observed, suggesting the potential for malfunction over time. It was essential to consider the hierarchical grouping. Figure 29 illustrates the relationship between the distances in the hierarchical clustering and the correlations [32].

It is now necessary to visualize the features that were produced following the completion of the preprocessing stage. It can be observed that the 15 sensors exhibit a discernible pattern. It was hoped that the model would be able to identify these patterns and provide a robust basis for further analysis, as illustrated in Figure 29. The covariance matrix demonstrates the degree of discrepancy between the two variables, whereas the correlation matrix illustrates the strength of their relationship. The correlation matrix below depicts the inter-feature relationships that were incorporated into the model. It is important to note the presence of red diagonals, which represent a correlation between a feature and itself. Additionally, there were instances of moderately negative correlations observed in the subsequent sensors.

In this technique, each data point is initially treated as an independent cluster. At each iteration, the clusters that are deemed to be similar are merged with other clusters until either one cluster or K clusters are formed. The color presentations by the groups highlight variables with analogous behaviors, thereby facilitating comprehension of the operational relationships of these sensors.

### 3.2. Methodology

As the data had been prepared in a base format, an algorithmic approach to solving the prediction problem could be selected. The choice of algorithmic method is dependent upon the quantity of data, the complexity of the problem, the final hardware to be used for processing, and other considerations. The data may be used in their current form to train a classifier for the actual class. However, to predict future classes based on the actual values, data must be shifted relative to the target to create a time gap.

The subsequent phase was the normalization of the data. It is essential to normalize data in cases where the input features exhibit disparate ranges in their amplitudes, as previously documented in the literature [33]. Otherwise, these values are misrepresented when machine learning methods are employed. The primary operation utilized by machine learning methods is multiplication within the functions. The multiplication of a large value results in an even larger value, which may be misinterpreted as importance. Scaling all values between, for example, [0, 1] is a fundamental aspect of a neutral comparison. In the context of classification, it is essential to one-hot-encode the target before training, to ensure accurate interpretation of the classes without misinterpreting the class order (0, 1, 2) as a ranking or importance.

The K-nearest neighbor (KNN) development process comprises three distinct stages: the network architecture, the activation functions, and the learning algorithm. During the training phase, the initial weights are treated as random variables. Once the first epoch has been completed, these values undergo a continuous, cyclical change. The KNN model was trained by dividing the random index input data into two groups, with the objective of effectively predicting a leak [34].

### 3.3. Modeling

This work employed an unsupervised learning technique to predict failures by training a K-means clustering model. K-means models construct n-clusters based on distances and group them together based on their proximity to the mean. As this is an unsupervised learning approach, cross-validation is not a straightforward option. To assess the performance and identify optimal parameters, the model was designed to create its own function, as follows.

The objective of the tested methodology was to determine the optimal K-means model parameters from the algorithm’s training data. The Elkan and Lloyd algorithms were employed for this purpose. Additionally, varying numbers of clusters were considered, including three, four, and five. Consequently, accuracy values of 0.6103, 0.3267, and 0.3552 were obtained for varying numbers of clusters, ranging from three to five. Thus, with an accuracy of 0.6103, three clusters was identified as the optimal set of parameters.

The number of neighbors to be used by default for neighbors queries was set to 3, 5, or 10. The weight functions used in the prediction were varied between uniform (in which all points in each neighborhood are weighted equally) and distance (in which points are weighted by the inverse of their distance). The algorithm was varied between BallTree and KDTree; the power parameter for the Minkowski metric was equivalent to using Manhattan distance (1) and Euclidean distance (2); and combinations were evaluated to determine the optimum KNN model in the following phase. The optimal parameters were determined to be the BallTree method, a power parameter equivalent to using Manhattan distance (1), a number of neighbors of 10, and uniform weights.

**Figure 30:**
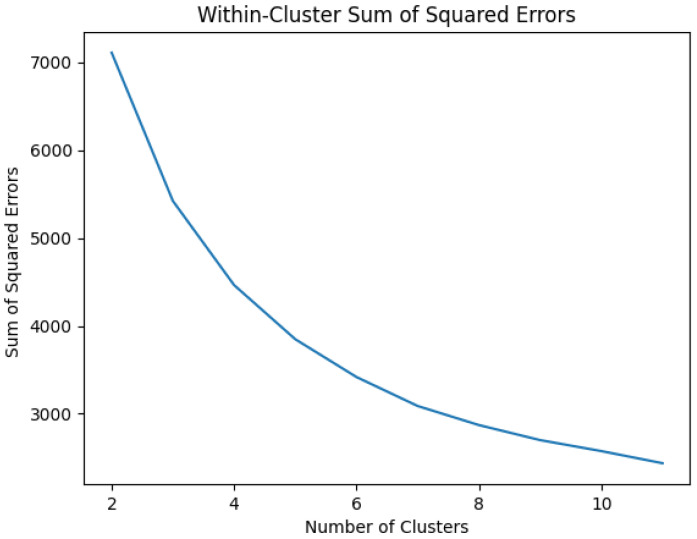
Data cluster.

It was crucial to visualize the errors in the K-Means model to ascertain the optimal number of clusters for training the model. Typically, one should seek an elbow shape and base the value on that. However, as illustrated in Figure 30, the plot lacks a defined elbow shape. Consequently, alternative methods were employed to determine the ideal number of clusters for the model.

The highest accuracy, 0.425, was achieved with any parameter values, provided that the number of clusters was 3. The proposed methodology utilized a three-cluster K-means model, and it was tested on the testing set. It is worth noting that the 42.5% accuracy rate was not optimal and was below the expected level of performance. Initially, the model had to undergo preprocessing of the test data in a manner consistent with that applied to the training data, as this provided the model with the best chance of success. This process involved splitting the data and then applying the preprocessing steps separately.

Subsequently, the K-means clustering algorithm (an unsupervised machine learning model) was employed to ascertain the locations of the failures. As K-means is an unsupervised learning model, the optimal number of clusters can be determined. To evaluate the performance of the models in a later stage, it is necessary to conduct some form of performance testing, such as accuracy, recall, and/or precision. In practice, the data labels can be identified through a process of permutation, which is a potential limitation of this technique. If there are more clusters than given labels, the model may not perform as well in terms of accuracy, recall, or precision.

Upon examination of the performance metrics, it becomes evident that the model was statistically balanced, as evidenced by the fact that the accuracy, precision, and recall were equivalent. This indicates that the model was capable of accurately classifying positive and negative samples with an equivalent rate of success. However, this may be misleading, as a model may perform poorly during training, and a significant number of zeros may be required to assess its performance during testing. In point of fact, the model exhibited a high degree of accuracy in predicting the 1 s and 2 s, which corresponded to the normal and recovering categories, respectively, based on the best labels function. This indicates that the model did not identify a single instance of “broken” in the test data. It is noteworthy that the model correctly identified a significant number of instances classified as “2”, which corresponds to the “normal” category. This observation suggests that the model demonstrated proficiency in detecting clusters that aligned with the expected norm. In comparison, the model exhibited limited capacity to identify instances classified in the “recovered” category. This finding indicates that the majority of the data remained classified as “normal”. It is plausible that the model could have consistently selected scenario 2 (normal data) and produced outcomes that were comparable.

## 4. Discussion

In regard to this subject, it was evident that an effective unsupervised learning model should be developed, and it was also necessary to evaluate its performance in comparison to other machine learning techniques. A supervised learning model, K-nearest neighbors, which is another clustering technique, was employed for comparison purposes. The preprocessed versions of the training and testing data were utilized for these models, with the same preprocessing and dimensionality reduction techniques applied. Subsequently, a clustering technique (K-nearest neighbors, KNN) was utilized to identify instances of failure (breakdowns) within a specified timeframe.

**Figure 31:**
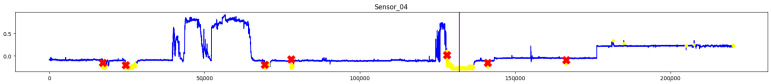
Sensor 04 data.

Following the creation of the K-means cluster model, a supervised learning model was constructed which also employed clustering techniques, including K-nearest neighbors (KNN). Two clustering models were selected for the practical application. The project now had a functioning KNN model and its predictions. To facilitate visualization, a single sensor (sensor 04) was utilized, as all normal, recovering, and broken occurrences were occurring simultaneously for all sensors (Figure 31).

Please direct your attention to the figure above. The purple vertical line demarcates the transition between the training set and the test set. The color yellow indicates the point at which the model estimated the recovery state, while the color red indicates an estimate of a pipe break, and the color blue indicates an estimated normal operation. As anticipated, the model exhibited robust performance on the left side (the training side). This is due to the fact that, in a supervised learning model, the model is provided with training data accompanied by labels, which enables it to correctly identify appropriate labels. However, an alternative outcome is observed when the test set is considered, situated to the right of the vertical line. In the initial stage, the model identified a recovery scenario (yellow), which was the intended outcome given the continued recovery from the previous spill. Moreover, the model predicted two instances of leak events (indicated by red Xs), which were anticipated based on the model’s training data.

**Figure 32:**
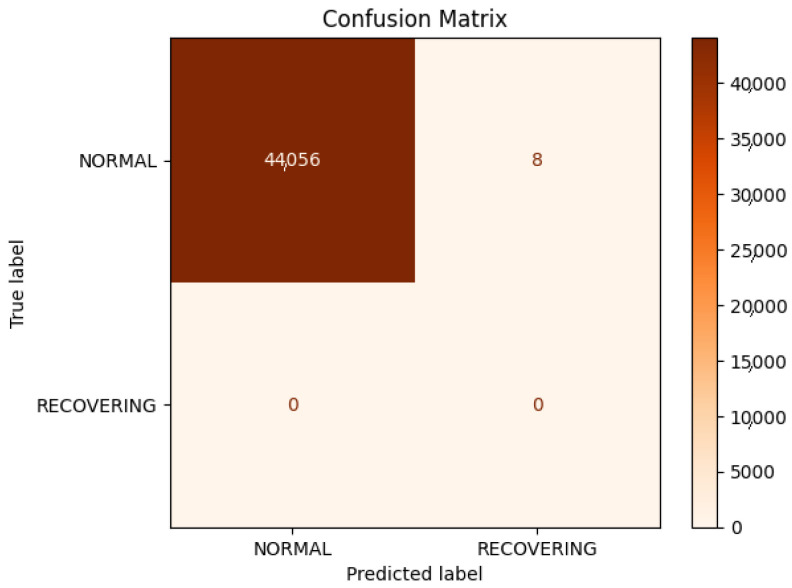
Confusion matrix.

The supervised learning model that was employed exhibited a high degree of similarity to the unsupervised model. As illustrated in Figure 32, the K-nearest neighbor (KNN) model, like the K-means model, only identified normal and recovering clusters. Both models appeared to encounter difficulties in identifying the data points to be clustered. It can be observed that the accuracy, precision, and recall were all equal, which suggests that the model was well-balanced under typical circumstances. However, the test accuracy, precision, and recall values were 0.9998, 0.9998, and 0.9998, respectively. The K-means model achieved a test accuracy of 0.9998, indicating that it was primarily guessing normal. It is probable that the model would have yielded comparable results had it solely guessed normal, given the pronounced imbalance in the data.

## 5. Conclusions

The initial step was to examine the dataset and represent it in a visual format. Although it is generally advised not to view all the data before dividing them into training and testing sets, for the specific purpose of this work and as a learning tool, it was decided to do so. Subsequently, the data were visualized in order to gain a more comprehensive understanding of the underlying patterns. Subsequently, the data were preprocessed to address the issue of missing data through the use of forward-fill propagation. A principal component analysis (PCA) identified the most pertinent features (columns) for incorporation during the training of the models. Subsequently, numerous K-means and K-nearest neighbor (KNN) models were trained and the optimal model was selected for each. Finally, the performance of the models was evaluated. In total, three models were utilized for the compilation process: two unsupervised models, namely PCA and K-means clustering, and one supervised model employing KNN.

In the K-means and K-nearest neighbor models, the accuracy, precision, and recall of the test appeared to be quite favorable, suggesting potential applications in supervisory data systems. It is crucial to underscore the necessity of conducting a thorough statistical analysis of the data and supplementary investigations to ascertain the degree of data balance. The majority of data were typically classified as normal, followed by a small proportion of instances in the leak recovery period and an even smaller number of leak occurrences. This suggests that the models may have encountered difficulty in distinguishing between cases that deviated from the norm (e.g., normal operation and leak recovery). It is imperative to be mindful of this phenomenon in practical scenarios. Moreover, for specific leak detection models, the categorization of hydraulic anomalies must be taken into account, due to their varying individual characteristics.

## Figures and Tables

**Table 1:** List of data types, variables, and quantities.

Variable	Quantity	Type
timestamp	220,320	datetime64[ns]
sensor 00	210,112	float64
sensor 01	219,951	float64
sensor 02	220,301	float64
sensor 03	220,301	float64
sensor 04	220,301	float64
sensor 05	220,301	float64
sensor 06	215,522	float64
sensor 07	214,869	float64
sensor 08	215,213	float64
sensor 09	215,725	float64
sensor 10	220,301	float64
sensor 11	220,301	float64
sensor 12	220,301	float64
sensor 13	220,301	float64
sensor 14	220,299	float64
sensor 15	0	float64
sensor 16	220,289	float64
sensor 17	220,274	float64
sensor 18	220,274	float64
sensor 19	220,304	float64
sensor 20	220,304	float64
sensor 21	220,304	float64
sensor 22	220,279	float64
sensor 23	220,304	float64
sensor 24	220,304	float64
sensor 25	220,284	float64
sensor 26	220,300	float64
sensor 27	220,304	float64
sensor 28	220,304	float64
sensor 29	220,248	float64
sensor 30	220,059	float64
sensor 31	220,304	float64
sensor 32	220,252	float64
sensor 33	220,304	float64
sensor 34	220,304	float64
sensor 35	220,304	float64
sensor 36	220,304	float64
sensor 37	220,304	float64
sensor 38	220,293	float64
sensor 39	220,293	float64
sensor 40	220,293	float64
sensor 41	220,293	float64
sensor 42	220,293	float64
sensor 43	220,293	float64
sensor 44	220,293	float64
sensor 45	220,293	float64
sensor 46	220,293	float64
sensor 47	220,293	float64
sensor 48	220,293	float64
sensor 49	220,293	float64
sensor 50	143,303	float64
sensor 51	204,937	float64
machine status	220,320	object

**Table 2:** Principal components analysis for sensors.

Relevance	Variable
1	sensor 04
2	sensor 50
3	sensor 27
4	sensor 36
5	sensor 29
6	sensor 17
7	sensor 28
8	sensor 35
9	sensor 30
10	sensor 32
11	sensor 48
12	sensor 33
13	sensor 26
14	sensor 23
15	sensor 16

## Data Availability

The codes implemented in this paper are available at https://ga-data-cases.s3.eu-central-1.amazonaws.com/pump_sensor.zip, accessed on 4 June 2024.
